# Case of Polypus Uteri, Removed by Excision: With Practical Remarks

**Published:** 1841-10

**Authors:** Chandler R. Gilman

**Affiliations:** Prof. of Obstetrics and the Diseases of Women and Children in the College of Physicians and Surgeons, New York


### Selections from American and Foreign Journals.

---

*Case of Polypus Uteri, removed by Excision: with practical remarks.* By Chandler R. Gilman, M. D., Prof. of Obstetrics and the Diseases of Women and Children in the College of Physicians and Surgeons, New York.—Polypus Uteri is a rare disease, and although it is a well settled principle, that the tumor should be removed as soon as possible, yet the manner of removal is still matter of dispute. The older English writers, are nearly unanimous in favor of the ligature, but among the more modern, Simson of Edinburg, Churchill of Dublin, Sir B. Brodie and Mr. Arnott, favor excision, and I find it stated in a late number of the British and Foreign Review, that it is now preferred by the majority of English practitioners. On the continent, excision has decidedly the majority in its favor. Dupuytren revived and established the practice in France, and in Germany, Osainder, Siebold and others have done the same; it is now everywhere preferred.

With all this weight of authority in its favor, I believe the operation has not been extensively adopted in America. I cannot find that it has ever been performed in New York. The fear of hæmorrhage, the authority of some of our most distinguished teachers, the habit of preferring the ligature, have each, probably, had their weight in producing this result. Believing that excision is, in the majority of cases, the better operation, I have thought that the report of a case in which it was practiced with the most entire success, might do good, by leading the junior members of the profession to examine the ground thoroughly before they resolve to "walk in the footsteps of their predecessors." Perhaps even some of the advanced may think proper to reconsider their opinions, and adopt an operation which, if it can be proved to be equally safe, is certainly in every other respect to be preferred.

*May* 29, 1841.—I was requested by Dr. M'Comb to see a German female, Mrs. M——, aged 33—married three or four months. Before seeing her, I received the following history of her case. Mrs. M. enjoyed very good health and was rather robust till about eight months ago, when the menses began to be very profuse, continuing for six or eight days, and very free during all that time. During the intervals, however, she had no sanguineous or other discharges. About two weeks after her marriage she was again profusely "*unwell*," and from that time to the present, the hæmorrhage has been constant, though at some times more profuse than at others. The general health is beginning to suffer seriously, the strength is gone, the appetite diminished, and the symptoms of anæmia are gradually being developed. Such was the brief history of the patient; her appearance verified it, the pallor is excessive, the pulse weak but easily excited, the breathing hurried, the muscular strength utterly prostrated. Ergot had been given a few hours before I saw her. The hæmorrhage was slight but she complained much of pain in the back, bearing-down efforts and frequent desire to make water. Bowels were moved freely by castor oil this morning, evacuations natural. *Examination per vaginam.*—The vagina is occupied at its upper part by a firm, dense tumor, about the size of a hen's egg: at first I supposed, from the woman's complaints, that it was very sensible to the touch; she screamed when I pressed it from side to side of the vagina. Passing the finger upward along the tumor, it was found to be attached to, or a continuation of, the anterior lip the os tincæ: the finger could easily be passed half round the tumor behind, and then into the os. The anterior lip was firmer than natural, though scarce so firm as the tumor: the posterior lip was natural, and the soft, spongy, relaxed feel which it presented, and which exactly resembled that of the os during the menstrual flow, contrasted remarkably with the dense cartilaginous feel of the tumor, and the anterior lip. Was this a polypus? The symptoms rendered it probable, but the examination was not quite satisfactory. The tumor was not pediculated, and it appeared to be sensible, at least pressure upon it gave pain. More careful examination cleared up the latter and the chief difficulty. I found by cautious trials, that tho' pressure upon the tumor gave pain, it was only when that pressure communicated motion to it; scratching, or even puncturing the tumor, (for I tried this by means of a pointed probe,) excited no sensation. This decided the diagnosis in favor of polypus, and the immediate removal of the tumor was resolved on. After a full consideration of all the particulars of the case, with the advantage of a consultation with Dr. Delafield, I resolved to remove it by excision. Upon the general merits of this operation and that by ligature, I shall remark by and by. The particular features of this case, which led me to prefer excision, were, the situation of the tumor, attached to the anterior lip of the os tincæ, and of course fully within reach; the density of its structure, rendering it probable that the process of decay would be slow and tedious; and the extreme heat of the weather, during which the constant presence for many days of a putrifying mass in the vagina, would be likely to induce serious symptoms. In addition to all these reasons, the fact that a certain number of cases of puerperal fever had appeared in New York during the last six or eight weeks, was not without its weight. It is well known that where peurperal fever has been epidemic, the operation of removing a polypus has been followed by a peritonitis, having all the characteristic symptoms of the puerperal epidemic. These considerations determined me to remove this tumor by the knife, which I accordingly did, in the presence and by the kind assistance of my colleague, Professor Parker.

The bladder and rectum having been well evacuated, Weiss' speculum vaginæ was introduced, and upon expanding the blades the tumor was brought fairly into view. The color was bright red, and it contrasted in this respect very strikingly with the vagina, which was singularly pale. I grasped the tumor with a pair of hooked forceps and tried to drag it down. I found, however, that here the speculum presented an obstacle, for as I pulled down, the ends of that instrument pushed up, or at least supported the parts. The speculum, too, hindered the free use of the fingers in the vagina; it was therefore withdrawn, and then the tumor was drawn down till the neck, or rather its junction with the anterior lip—for the tumor was not pediculated—was fairly within the reach of the finger. Cooper's bistoury, with blunt point and a small portion of cutting edge, was now introduced flat upon the fore-finger, and when the point had fairly passed the base of the tumor, a few strokes sufficed to divide it. On withdrawing the polypus in the grasp of the forceps, I was prepared for some hæmorrhage, and placed a napkin over the vulva; to my surprise, on looking at it after the lapse of several minutes, it was not even tinged. Before I left the room there was a very slight stain, but nothing worthy of the name of hæmorrhage occurred then or since. The discharge ceasing with the removal of the tumor, the recovery was uninterrupted and satisfactory. On examining the tumor, it was found to consist of dense white fibrous matter, presenting upon its very surface some traces of vascularity, but within entirely devoid of anything of the kind.

*Remarks.*—There are two or three points about this case to which I wish to direct the reader's attention. 1st. The fact that though the tumor was insensible, yet when pressure communicated motion to it, great pain was complained of. The pain in this case was doubtless caused by the forcible dilatation of the cervix when the tumor was moved. May not something of this sort have given rise to the opinion that tho' polypi are very generally not sensible, yet in some rare cases they are acutely so? I throw out the idea merely as a suggestion. As an aid to diagnosis, puncturing the tumor with a pointed probe, or scratching it with the sharp finger nail, should be remembered. 2nd. The bright florid red of the tumor, when the vaginal mucous membrane was remarkably pallid. This certainly depended on the lining membrane of the tumor. What is that lining membrane, and has it any connexion with the profuse hæmorrhages by which these cases of polypi are distinguished? The source of these hæmorrhages has long been a disputed point, and is yet involved in the most profound obscurity. Gooch, believes, that the flow is from the lining membrane of the tumor. Hamilton contends, that to support the growth of the tumor, a sanguineous flux takes place to the uterus. Levret refers it to the distension of the uterus, the enlargement and opening of its venous sinuses. "Hence,"—says he—and the remark is quoted with approval by Boivin and Duges—"polypi of the cervix seldom cause hæmorrhage." In this conflict of opinion, I confess I incline to that of Gooch, and I am somewhat strengthened in my opinion, by the appearance of great vascularity by which the surface of this polypus was distinguished, as well from the cervix uteri as from the vagina. At any rate, the fact of vascularity is worth noting. 3rd. As to the operation, the directions in the books are, to grasp the tumor, draw it down till the stalk pass out of the vagina, then with a bistoury or scissors cut it off close to the vulva. This, as I happen to know, was the practice of Lisfranc. Now to this dragging down I object, as inflicting both unnecessary pain and additional danger on the patient, and being quite unnecessary. That it gives very great pain will readily be believed, but it is not equally certain that it is attended with danger? When we thus pull the uterus down, upon what does the strain fall? certainly upon the reflexions of peritoneum, forming the broad, the utero-sacral, and the utero-vesical ligaments. Now, this peritoneum is slightly, if at all, extensible, and therefore can only give way by being detached at some point or points. Is not this violence offered to the peritoneum exactly the means most likely to produce the thing we have chiefly to fear after this operation, I mean peritonitis? But is this dragging the polypus out of the vulva, necessary? I think not. If the pedicle is fairly within the reach of the fingers at the point where we wish to divide it, and we use a bistoury with blunt point and little cutting edge, especially if the blade have some lateral curvature, the operation can be performed without much difficulty; and, supposing the operator to possess a reasonable share of caution and dexterity, without the slightest danger of wounding either the cervix or the vagina. I had intended to offer some observations on the comparative merits of excision and ligature, but my paper has already extended to sufficient length, and I must defer them.—*N. Y. Med. Gaz.*

